# Contribution to the Evaluation of Physicochemical Properties, Total Phenolic Content, Antioxidant Potential, and Antimicrobial Activity of Vinegar Commercialized in Morocco

**DOI:** 10.3390/molecules27030770

**Published:** 2022-01-25

**Authors:** Mohammed Kara, Amine Assouguem, Mohamed El Fadili, Safaâ Benmessaoud, Samar Zuhair Alshawwa, Omkulthom Al Kamaly, Hamza Saghrouchni, Abdou Rachid Zerhouni, Jamila Bahhou

**Affiliations:** 1 Laboratory of Biotechnology, Conservation and Valorisation of Natural Resources (LBCVNR), Faculty of Sciences Dhar El Mehraz, Sidi Mohamed Ben Abdallah University, Fez 30000, Morocco; safaa.benmessaoud@usmba.ac.ma (S.B.); abdourachid.zerhouni@usmba.ac.ma (A.R.Z.); jamila.bahhou@usmba.ac.ma (J.B.); 2 Laboratory of Functional Ecology and Environment, Faculty of Sciences and Technology, Sidi Mohamed Ben Abdellah University, Fez 30000, Morocco; assougam@gmail.com; 3 Laboratory of Engineering Materials Modeling and Environmental, Faculty of Sciences Dhar El Mahraz, Sidi Mohamed Ben Abdellah University, Fez 30000, Morocco; mohamed.elfadili@usmba.ac.ma; 4 Department of Pharmaceutical Sciences, College of Pharmacy, Princess Nourah bint Abdulrahman University, P.O. Box 84428, Riyadh 11671, Saudi Arabia; SZAlshawwa@pnu.edu.sa; 5 Department of Biotechnology, Institute of Natural and Applied Sciences, Çukurova University, Adana 01250, Turkey; hsaghrouchni@student.cu.edu.tr

**Keywords:** vinegar, polyphenols, fermented fruits, antioxidant activity, antimicrobial activity, bioactive molecules

## Abstract

Vinegar is a natural product widely used in food and traditional medicine thanks to its physicochemical properties and its richness in bioactive molecules. However, its direct use by consumers can have complications and undesirable effects. Therefore, this study contributes to investigating the physicochemical and biological properties of eleven vinegars marketed in Morocco. Determination of pH, acetic acid, conductivity, total soluble solids and alcohol content in vinegar was carried out. The polyphenols (TP), flavonoids (TF), and condensed tannins (CT) content was determined, and their antioxidant activities were evaluated using 2,2-diphenyl-1-picryl Hydrazyl (DPPH), Ferric Reducing Antioxidant Power (FRAP) and Phosphomolybdenum Reduction Assay (TAC). Then, the antimicrobial activity was studied against four pathogenic bacteria and two fungal strains, using the disk diffusion and the microdilution method. This study showed a wide range of acetic acid values from 0.65 ± 0.29 to 5.15 ± 0.20%. The high value of TP, TF, and CT in our samples V10, V9, and V4 was 655.00 ± 22.2 µgGAE/mL, 244.53 ± 11.32 µgQE/mL and 84.63 ± 1.00 µgTAE/mL, respectively. The tested strains showed variable sensitivities to the different samples with inhibition zones ranging from 6.33 ± 2.08 to 34.33 ± 0.58 mm. The lowest minimum inhibition concentrations were recorded against *Staphylococcus aureus* ATCC29213 ranging from 1.95 to 7.81 µL/mL. While *Aspergillus niger* ATCC16404 showed resistance against all of the analyzed samples. In general, vinegar commercialized in Morocco presents a variable range of products with variable properties. Indeed, must take into account this diversity when using it. A future study is needed to identify the phytochemical composition that will further the comprehension of this variability and contribute to its valorization.

## 1. Introduction

Around the world, the use of vinegar is becoming increasingly important. It is used in various fields of application such as the nutritional, medicinal, and pharmaceutical fields. Its richness in biomolecules allows it to be a product of preference. Vinegar is a condiment that can be found throughout the market [1,2,3,4]. It can be made from various fruits containing carbohydrates and is obtained by alcoholic fermentation and oxidation, leading to the formation of acetic acid. This production is performed with traditional or industrial fermentation processes [5]. In the traditional method, the raw material is fermented with spontaneous microorganisms, taking weeks or months, while the industrial method is carried out to produce a fast fermentation using a liquid of fruits submerging the bacterial starter culture [3,6]. The final product of vinegars produced by different methods differs in its profile. In other words, there are various factors influencing the physicochemical and phytochemical parameters of the final product of vinegar, such as the raw material, production methods, temperature, pH and microorganisms involved in the fermentation process [7,8,9]. In fact, this variability influences its pharmaceutical and medicinal effects [3].

Several research studies have reported that the effect of vinegar on the human body is related to its phytochemical composition and its concentration [4], which provides excellent biological activity. According to Ozturk et al. [6], almost all of the traditional and industrial vinegar samples showed antibacterial activity at varying levels. The vinegar was shown to be effective as an antimicrobial agent against several foodborne agents and pathogenic strains such as *Klebsiella pneumoniae*, which causes community-onset infections and *Escherichia coli*, *Bacillus cereus*, *Salmonella typhi*, *Pseudomonas aeruginosa* and *Staphylococcus aureus* responsible for diarrhea [10,11,12,13,14]. The antifungal activity of vinegar was tested against various fungal strains of *Aspergillus*, *Fusarium*, and *Candida* [15,16]. Indeed, the acetic acid content in vinegar confers it an activity which can be used for the treatment of various infections even at low concentrations [3,17]. In addition, vinegar was used recently in certain dietary behaviors to prevent non-severe and severe acute respiratory syndrome (SARS) caused by a coronavirus (SARS-CoV) or as an effective disinfecting agent to stop its transmission. Several studies reported that acetic acid content was responsible for this antivirus effect by its application alone or combined (at 0.34% concentration) with hydroxychloroquine [18,19,20]. The polyphenol content in vinegar is known for its considerable biological effect on the human body as an antioxidant agent involved in several biological processes [4,21]. The major phenolic compounds that can be found in vinegar are phenolic acids, flavonoids, tannins, anthocyanins, and stilbenoids [22,23,24]. In addition, vinegar is rich in mineral and volatile compounds [6]. These properties of vinegar mean it experiences remarkably widespread consumption in the whole world. This leads us to pay particular attention to the quality of the vinegar produced and to question the products available on the market. Therefore, the present study aimed to investigate the variation of physicochemical parameters in different kinds of vinegar commercialized in Morocco. Meanwhile, we characterized the total phenolic content, total flavonoid, and condensed tannins and their antioxidant and antimicrobial activities. This study is considered the first to be conducted in Morocco and would be helpful to characterize different kinds of Moroccan vinegar in order of its valorization in the future. 

## 2. Results and Discussion

### 2.1. Physicochemical Analysis

The results of the physicochemical analysis of vinegar commercialized in Morocco are shown in Table 1. The pH measurement showed variable values ranging between 2.37 ± 0.09 and 4.47 ± 0.08 for V4 and V8, respectively. In general, pH values of traditionally produced vinegar were higher than the industrially produced ones, and many other samples. These results are in line with previous studies established by [25,26,27]. The TSS values were between 1.03 ± 0.18 °Brix and 8.67 ± 0.12 °Brix. The V9 and V8 values represent higher conductivity values in the order of 6.19 ± 0.29 and 5.67 ± 0.50 µS/cm, respectively. Compared to the other research, these values are similar to those reported by [14,28,29,30]. The acetic acid contents in vinegar V2 (5.15%), V9 (4.98%), V1 and V5 (3.75%) are higher than those of the other samples. The alcohol content in the vinegar samples of this research ranged from 0.03 ± 0.02 to 1.00 ± 0.00% for V2 and V3, respectively. Except for V2, these values were not in conformity with the standard which must be less than 0.1% for residual alcohol content and more than 5% for the degree of acidity [31].

### 2.2. Determination of Total Phenolic, Total Flavonoids, and Condensed Tannins Content 

Polyphenols and flavonoids are the main bioactive compounds in vinegar that can be obtained from various raw materials, which are responsible for several positive effects on health [32,33]. Table 1 summarizes the results of the phenolic compounds in the different types of vinegar studied. It was demonstrated that V10, V7, V9 are very rich in total polyphenols (655.00 ± 22.2, 577.89 ± 13.47 and 521.22 ± 12.73 µg GAE/mL, respectively) compared to the other samples. The lowest value was 6.22 µg GAE/mL, which was recorded in V6. The flavonoid content range between 18.67 ± 4.56 and 244.53 ± 11.32 µg QE/mL in all samples except V6 and V11, which do not show any traces of flavonoids. The highest concentration of Flavones and Flavonols recorded in our vinegar samples was 225.20 ± 17.6 µg QE/mL for V9, followed in descending order by V7 with 114.72 ± 11.16 µg QE/mL, while the lowest values were 2.33 ± 0.58 µg QE/mL and 3.67 ± 3.50 µg QE/mL for V2 and V3, respectively. These results are in line with previous research that reported that TPC and TFC in apple vinegar are lower than in most fruit vinegar [6,12,14,34]. Sengun et al. reported that the TPC in grape and apple vinegar was 1025 ± 2.83 and 988 ± 2.83 mg GAE/L, and the TFC was 221.81 ± 3.43 and 174.79 ± 3.40 mg Catechin/L, respectively [14]. Yun et al. found that TPC and TFC contained in peach were 437.6 ± 29.8 mg/L and 30.3 ± 2.1 mg/L, respectively [35]. Moreover, the total phenolic content (TPC) and total flavonoid content (TFC) of traditional vinegar are significantly higher than those of industrial vinegars [6,12]. In the case of condensed tannins, the concentration varies in all the studied samples between 84.63 ± 1.00 µg TAE/mL and 0.69 ± 0.53 µg TAE/mL. V6 record the lowest concentration, while V4 has a very high concentration compared to the other samples. According to the scientific literature, procyanidins and condensed tannins are the major substances of most apple varieties [36]. In general, the phytochemical compound in vinegar varies widely depending on the raw material used in its production [6] and on the strain of yeast and acetic acid bacteria involved [9].

### 2.3. In Vitro Antioxidant Activity

**Figure 1:**
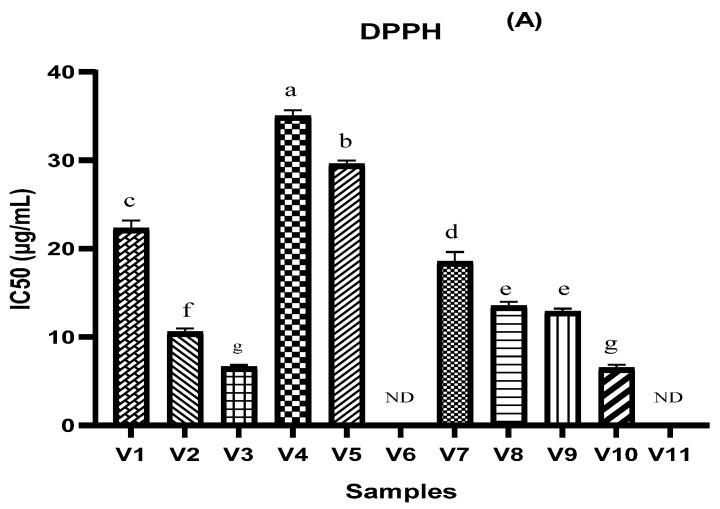

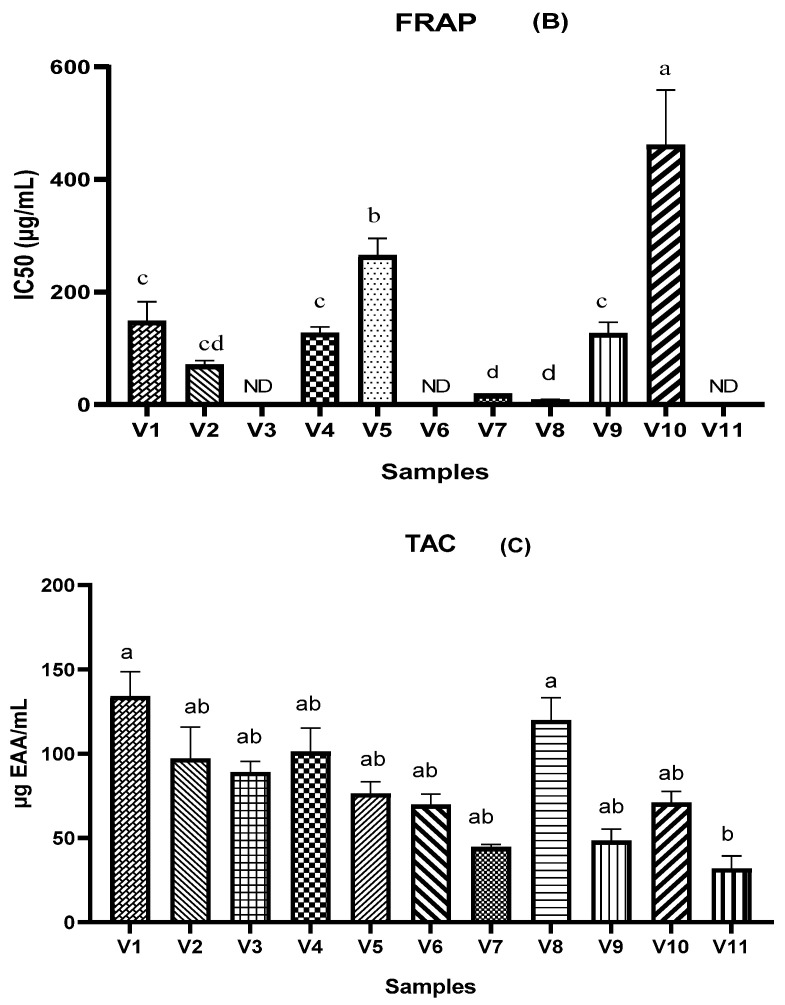
Antioxidant activity of different vinegar samples tested by 2,2-diphenyl-1-picryl hydroxyl radical scavenging assay (**A**), ferric reducing antioxidant power assay (**B**), and phosphomolybdenum reduction assay (**C**). ND = 50% inhibition not determined. Values that do not share the same letter are significantly different.

The antioxidant activity of the different types of vinegar studied in this research was evaluated using three complementary tests: DPPH, FRAP, and TAC assay. The results are shown in Figure 1. It is shown in these results (Figure 1A) that V10, V3, V2, V9, and V8 have a lower DPPH IC50 value (6.441 µg/mL, 6.581 µg/mL, 10.508 µg/mL, 12.852 µg/mL, and 13.465 µg/mL, respectively), while 50% inhibition for V6 and V11 was not attained. According to the results found by [35] on various fruit vinegars, the peach vinegar and the grape vinegar record a higher value of antioxidant activity using DPPH assay (94.7 ± 0.7% and 82.4 ± 1.5%, respectively) in comparison with apple vinegar which was between 61.2 ± 0.4% and 72.2 ± 1.4% [35]. In addition, Ozturk et al. reported several ranges of DPPH values, which ranged from 4.93% to 89.53% for grape vinegar, while they ranged from 0.53% to 65.12% for apple vinegar [6]. In addition, the antioxidant property of fruit vinegar, including traditional balsamic vinegar and peach vinegar, was over 90% [35]. The antioxidant power based on the FRAP assay indicated significant outcomes between different samples (Figure 1B). The IC50 values of V8, V7, and V2 were much lower (8.369 µg/mL and 19.072 µg/mL, respectively) than V1, V4, V5, V9, and V10 while the highest IC50% value was recorded in the V10 sample (461.536 µg/mL). According to [12], the FRAP values of apple vinegar were lower than those of grape vinegar. As shown in Figure 1C, among the tested vinegar samples, V1 and V8 were found to exhibit the highest total antioxidant capacities with the values of 134.068 and 119.903 µg AAE/mL, respectively. While the lowest values were recorded in V11 (31.814 µg AAE/mL). In a related study, [35] recorded that, in peach vinegar, the IC50 was (13.1 ± 0.6 µg/mL). Several studies have reported that the difference in antioxidant capacity is due to differing their phytochemical profiles and initial raw materials [14].

### 2.4. Antimicrobial Analysis 

#### 2.4.1. Disk Diffusion Assay

The antimicrobial activities of the vinegar samples were tested against bacterial and fungal strains using the disc diffusion method, and the outcomes are shown in Table 2. The sensitivity of the bacteria to the various vinegar samples was highly variable. The highest value of inhibition was recorded against *K. pneumonia (ESBL-KP)* in the range of 8.67 ± 1.15 mm and 32.67 ± 2.52 mm for V11 and V5, respectively. The V11 and V10 revealed a higher antibacterial activity (25.67 ± 0.58 and 24.67 ± 0.58 mm) against the *E. coli* ATCC strain followed by V9, V3, and V2 with DIZ of 18.67 ± 2.31, 18.33 ± 1.53 and 18.00 ± 0.00 mm, respectively., while the lowest activity was 7.33 ± 0.58 and 6.67 ± 0.58 mm for V4 and V6, respectively. Except for sample V11, we can note that all samples have antibacterial activity against *E. coli* CIP ranging between the lowest value of 6.33 ± 2.06 mm and the highest of 25.33 ± 0.58 mm for V6 and V10, respectively. Antibacterial activity against *S. aureus* was shown to be the lowest in this study with DIZs ranging from 9.00 ± 1.73 mm to 16.33 ± 1.15 mm for V4 and V9, respectively. Concerning *A. niger* strains, we can clearly note its resistance to all the vinegar samples used in this study, while the antimicrobial activity against *C. albicans* yeast ranged between 8.00 ± 0.00 and 34.33 ± 0.58 mm for V7 and V1, respectively. Generally, the peach, grape, and apple vinegar purchased from cooperatives and ACV obtained from the local market had a stronger antibacterial activity. Several studies showed that apple vinegar had low antimicrobial activity against almost all strains of microorganisms [16,37,38]. The antimicrobial activity of each vinegar sample is strongly correlated with their phytochemical compounds [4,39]. Indeed, the qualitative and quantitative difference of organic acids, polyphenols and primary metabolites contained in each kind of vinegar are the main factors responsible for the variation of the antimicrobial activity [40,41]. The presence of bioactive compounds, such as Gallic acid, Epicatechin-3-gallate, Caffeic acid, Catechins, amino acids and acetic acid, in the vinegar can inhibit the bacteria strains at low concentration such as *S. aureus*, *S. mutans*, *E. coli* O157:H7 and *P. aeruginosa* [40,41]. The previous literature showed that polyphenols contained in the apple vinegar is generally lower than that found in the other fruits’ vinegar [42,43].

#### 2.4.2. Determination of the Minimum Inhibitory Concentration (MIC)

The results of the antimicrobial activity of the samples obtained through the microdilution method were presented in Table 2. The Minimum Inhibitory Concentration (MIC) recorded in this study was in the range of 1.95 µL/mL and 500 µL/mL. The MIC values recorded against *E. coli* ATCC were 1.95 µL/mL for (V6 and V9), 3.9 µL/mL for V8, and 7.81 µL/mL for the other samples. The lowest antimicrobial activity recorded against *E. coli* CIP was 62.5 µL/mL by V7 sample and the highest was 1.95 µL/mL. The antimicrobial activity of all samples against *S. aureus* ranged between 1.95 µL/mL and 7.81 µL/mL. Generally, the lowest antimicrobial activity in this study was recorded against the yeast *C. albicans* which falls within the range of 31.25 µL/mL and 500 µL/mL for V6 and V5, respectively, and the highest activity was against *S. aureus* with MIC of 1.95 µL/mL. According to Yagnik et al., the MIC for *C. Albicans* was required at 1/2 ACV and for *S. aureus* was 1/25 dilution ACV was required, whereas for *E. coli* cultures the value was 1/50 ACV dilution [16]. As reported by Sengun et al., apple vinegar had lower antimicrobial activity (MIC=12.5–25%, *v*/*v*) than that recorded in grape vinegar (MIC=3.12–6.25%, *v*/*v*) against all studied bacterial strains. In general, apple vinegar had higher inhibitory concentrations than grape vinegar [14]. 

#### 2.4.3. Principal Component Analysis of Various Studied Parameters 

**Figure 2:**
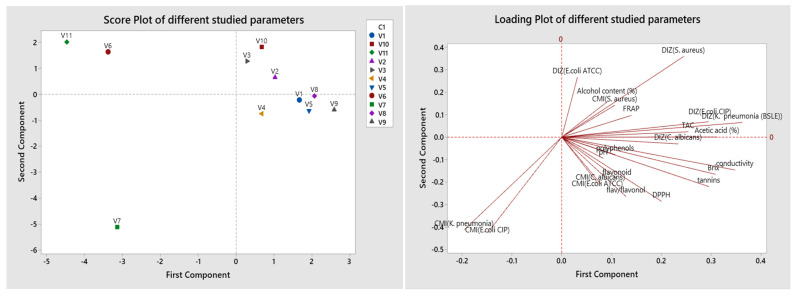
Principal component analysis (PCA) of the various commercialized vinegar samples using the assessed parameters.

Results of PCA obtained in Figure 2 show that the eigenvalues of the first four principal components represent 73.1% of the variation in the data. The first principal component shows a strong positive correlation with acetic acid, Brix, conductivity, and DIZ of *K. pneumonia*. The second component shows a positive correlation with DIZ of *S. aureus*, and a negative association with MIC of *E. coli* CIP and MIC of *K. pneumonia*. While the third component is correlated positively with flavonoids and flavonols/flavanone, and negatively with MIC of *E. coli* ATCC and C. *albicans*. The fourth one shows a negative correlation with polyphenols, and positively correlated with pH and MIC of *S. aureus*. In general, the variables are distributed on the different sides of the axis. However, the projection of the scoring diagram and the contribution diagram visually shows a positive contribution of physicochemical properties on the first main axis in positive correlation with V1, V2, V5, V8, and V9, while V6 and V11 were correlated negatively. The second component measures some of the antimicrobial activity of V3, V7, and V10. 

We could conclude, based on these data, that the vinegar produced by the cooperatives and those obtained from peach, quince, and grapes have important physicochemical and biological properties. The samples obtained from the herbalists show variable characteristics. In general, the vinegar marketed in Morocco presents a variable range of products with variable properties. According to several studies, the therapeutic effects of vinegar are highly due to its bioactive compounds content [44,45,46,47,48,49]. Apple vinegar is highly rich in organic acids, phenolic acids, tannins, flavonoids, and carotenoids, which confer it with a high level of antioxidants and antibacterial properties. For a reasonable application of vinegar, its use by consumers must take into account this diversity of product characteristics.

## 3. Materials and Methods

### 3.1. Raw Material

Eleven commercial kinds of vinegar were used in this study. Ten of these were traditionally prepared from apple fruits (V1, V3, V4, V5, V6, V10, and V11), quince (V7), peach (V8), and grape (V9), and one sample was industrially prepared from apple cider (V2). The V2 and V9 samples were purchased from the local supermarket of Fez, Morocco, while V3 and V5 were obtained from vinegar-producing cooperatives located in Imouzzer Kander, Morocco, and V10 from cooperative located in Sefrou, Morocco. The other samples (V1, V4, V6, V7, V8, and V11) were purchased from Fez herbalists. All samples were centrifuged to reduce the turbidity and were stored at 4 °C for later use.

### 3.2. Physicochemical Analysis

The percentage of acetic acid in the samples was calculated using NaOH (0.1 mol/L) [50]. The pH value was determined using a previously calibrated pH Meter SELECTA pH-2005 (SOMESTIM, Rabat, Morocco). Conductivity (µS/cm) was measured by direct reading using a previously calibrated conductivity meter SELECTA CD 2005 (Lab Associates, Oudenbosch, Netherlands). A handheld refractometer with Automatic Temperature Compensation (ATC) Model ATAGO Pocket Refractometer (ATOGO, Fujian, China) was used to measure the total soluble solids (TSS) (°Brix) and alcohol content (%) of the samples [51].

### 3.3. Determination of Total Phenolic Content (TPC) 

The TPC of the samples was determined using the method described by [52,53]. Briefly, 0.1 mL of Folin–Ciocalteu reagent (25%) is mixed with 0.1 mL of vinegar. The mixture is stirred vigorously and 2 mL of 2% sodium carbonate was added. After 30 min of incubation at room temperature, spectrophotometry measurement (SOMESTIM, Rabat, Morocco) was applied to the set at 760 nm. The results are expressed as µg GAE/mL of vinegar sample using standard concentration curve y = 0.0006x + 0.0936, R² = 0.94.

### 3.4. Determination of Total Flavonoids Content (TFC) 

Total flavonoids content was determined by the methods previously described by [54]. In a test tube, 1 mL of vinegar and 1 mL of AlCl_3_ (2%) methanol solution were mixed. The absorbance of the set was measured by a UV-visible spectrophotometer UV-1600PC (VWR, Fontenay-sous-Bois, France) at 430 nm after 15 min of incubation. Ethanol and AlCl_3_ solution served as a control. All experiments were conducted in triplicate. A quercetin calibration curve (y = 0.0149x + 0.0528, R² = 0.99) was used to determine flavonoid concentration as micrograms of Quercetin Equivalent per mL of vinegar (µg QE/mL).

### 3.5. Determination of Flavones/Flavonols Content

Flavone and Flavonol contents of the samples were determined by mixing 0.5 mL of vinegar with 1.5 mL of ethanol, 0.1 mL of AlCl_3_ (10%) methanol solution, 0.1 mL of (CH_3_COO, Na), and 2.8 mL of distilled water in a test tube. The set was incubated for 30 min at room temperature. Absorbance of the set was measured at 415 nm using a UV-visible spectrophotometer UV-1600PC (VWR, Fontenay-sous-Bois, France). Our samples’ concentration of flavones and flavonols was calculated using the calibration curve obtained using Quercetin as the standard (y = 0.0019x + 0.0447, R² = 0.98). All operations were repeated three times [55].

### 3.6. Determination of Condensed Tannins Content (CTC) 

Calorimetric testing as described by [56] was used to determine the tannin content of the samples. Briefly, 100 µL of the sample was mixed with 500 µL of Folin–Ciocalteu and 1 mL of sodium carbonate (7.5%). The absorbance was measured at 760 nm after 30 min of incubation. The values of condensed tannins of vinegar were expressed as micrograms of Tannic Acid Equivalent per mL of vinegar (µg TAE/mL of vinegar) using standard concentration curve y = 0.0051x + 0.0289, R^2^ = 0.99.

### 3.7. The 2,2-Diphenyl-1-picryl Hydrazyl Radical Scavenging Activity of Vinegar

The antioxidant activity of the samples was determined using DPPH assay as described by [57]. This method involves mixing 100 μL of each methanol solution of the tested vinegar samples with 750 μL DPPH in methanol (0.004%). Its optical density is then determined at 517 nm after incubation at laboratory temperature for 30 min. Methanol serves as a blank control. The following equation was used to determine the percentage inhibition of DPPH:

PI (%) = (1 − (A_c_/A_s_))∗100 (1)

where A_c_ = the absorbance of the control sample and A_s_ = the absorbance of the tested sample. The half-maximal inhibitory concentration (IC50) was determined graphically. 

### 3.8. Ferric Reducing Antioxidant Power of Vinegar

This test was performed using the method described by [58]; briefly, 500 μL of phosphate buffer (0.2 M; pH = 6.6) and 500 μL of potassium ferricyanide (1%) were added to 100 μL of the sample at different concentrations prepared in methanol. After 20 min incubation at 50 °C in a water bath, 500 µL of aqueous TCA (10%) solution, 100 μL FeCl_3_ (0.1%), and 0.5 mL of distilled water were added to the reaction medium. The absorbance of the resulting preparation was determined by the colorimetric method using UV-visible-spectrophotometer UV-1600PC (VWR, France) at 700 nm. The tubes containing all the reagents except samples were used as a blank test. Determination of IC50 reflects the concentration of antioxidants required to obtain an absorbance of 0.5 nm. Higher absorbance indicates the higher reducing power of the sample. Assays were carried out in triplicate [58].

### 3.9. Phosphomolybdenum Reduction Assay of Vinegar

Determination of the total antioxidant capacity (TAC) of the samples was conducted by mixing 25 µL of the sample with 1 mL of liquid reactive solution (0.6 M sulfuric acid, 28 mM sodium phosphate, and four mM ammonium molybdate). Then, the mixture was incubated for 90 min in a water bath at 95 °C. The absorbance of the incubated mixture was determined using a spectrophotometer UV-1600PC (VWR, Fontenay-sous-Bois, France) at 695 nm absorbance. The antioxidant capacity was expressed as micrograms of ascorbic acid equivalent per mL of vinegar (µg AAE/mL of the sample). The equation of the standard concentration curve was y = 10.761x + 0.248, R² = 0.98. Methanol was used instead of the sample as a negative control [59].

### 3.10. Antimicrobial Analysis

#### 3.10.1. Microbial Strains and Inoculums Standardization

Six microbial strains were used in this study. Four bacterial strains: *Escherichia coli* ATCC 25922, *Escherichia coli* CIP 53126, *Klebsiella pneumonia* (ESBL-KP) were Gram-negative, and the one Gram-positive strain was *Staphylococcus aureus* ATCC 29213. *Candida albicans* ATCC 10231 and *Aspergillus niger* ATCC 16404 were used as fungal strains. All microbial strains were provided from the Microbiology Laboratory, Faculty of Medicine and Pharmacy of Fez, Morocco. The cultures were stored on Muller–Hinton agar at 4 °C. The different microbial strains were standardized and inoculated following the method described in [60] for bacteria and [61] for fungal strains.

#### 3.10.2. Disk Diffusion Assay

Disk diffusion assay (DD), based on the Kirby–Bauer method of [62] was used to determine the antimicrobial activity, with slight modification. The standardized suspension (1–5 × 10^8^ CFU/mL) of the previously prepared isolates were inoculated onto Mueller–Hinton agar (MHA) for the bacteria strain. Whatman paper discs (6 mm) impregnated with 20 µL of the vinegar samples were gently placed onto the surface of the pre-inoculated agar. The plates were left to dry for 10 min, after which they were incubated for 24 h at 37 °C [40]. After incubation, the diameters of the inhibition zones were measured in mm. Fluconazole, Ampicilline, Streptomycine and voriconazole were used as positive controls.

#### 3.10.3. Determination of the Minimum Inhibitory Concentration

The minimum inhibitory concentration (MIC) of the samples was determined using microdilution assays according to the standards of the NCCLS [63], with slight modification. Under sterile conditions, ten concentrations ranging from 500 to 3.91 µL/mL of each vinegar sample were prepared by successive two-fold dilutions in distilled water. Then, 50 μL of the culture medium MH broth was deposited into each well of the microplate, while the first and the last wells were devoted to a negative control containing 100 μL of the vinegar and positive growth control, respectively. Microdilutions were made by transferring 50 μL by a factor of ½ into each well. The microplate was then inoculated with 50 μL of the microbial suspension. The inoculated microplate was incubated for 24 h at 37 °C for bacteria, and 25 °C for fungal strains. The colorimetric method based on the reagents of 2,3,5-triphenyltetrazolium chloride (TTC) was used to read the results. After 2 h of incubation, the MIC was determined as the lowest concentration that does not produce a pinkish coloration where there is growth due to the activity of the dehydrogenases [40].

### 3.11. Statistical Analysis

The experiments were conducted in triplicate. Multiple comparison and separation of the mean values was performed by a post hoc Tukey test at *p* < 0.05. Principal component analyses (PCA) were accomplished using Minitab19.1 software to classify each kind of vinegar’s in relationship with the parameters analyzed.

## 4. Conclusions

This study contributes to evaluating the physicochemical, biochemical properties, antioxidant potential, and antimicrobial activity of different kinds of vinegar commercialized in Morocco. The results showed a large diversity of vinegar products intended for direct use by the consumer. The high values of phytochemical were 655.00 ± 22.2 µg GAE/mL for TPC, 244.53 ± 11.32 µg QE/mL for TFC, and 84.63 ± 1.00 µg TAE/mL for CTC in V10, V9, and V4, respectively. The strains tested showed variable sensitivities to the different samples studied with inhibition zones ranging from 6.33 ± 2.08 mm to 34.33 ± 0.58 mm. The lowest minimum inhibition concentrations (MIC) were recorded against *Staphylococcus aureus* ATCC 29213 ranging from 1.95 to 7.81 µL/mL of vinegar, while the filamentous fungi strain studied showed resistance against all of the analyzed samples. Therefore, the application of vinegar must take into account its phytochemical characteristics. A future study is needed to identify the phytochemical composition that will better elucidate this variability and contribute to its valorization.

## Figures and Tables

**Table 1:** Physicochemical properties and bioactive compounds of different vinegar samples.

Samples ID	Acetic Acid (%)	pH	TSS (°Brix)	Alcohol Content (%)	Conductivity (µS/cm)	TPC µgGAE/mL	TFC µgQE/mL	Flavones et Flavonols µgQE/mL	CTC µgTAE/mL
V1	3.75 ^b^ ± 0.14	3.33 ^b^ ± 0.14	5.30 ^de^ ± 0.14	0.50 ^bc^ ± 0.10	3.78 ^b^ ± 0.25	278.40 ^e^ ± 21.4	131.09 ^c^ ± 4.06	67.18 ^d^ ± 8.84	44.12 ^d^ ± 0.74
V2	5.15 ^a^ ± 0.20	2.60 ^de^ ± 0.10	4.96 ^e^ ± 0.10	0.03 ^d^ ± 0.02	3.41 ^bc^ ± 0.16	480.67 ^c^ ± 16.91	21.56d ^de^ ± 5.77	2.33 ^e^ ± 0.58	53.91 ^b^ ± 1.36
V3	2.82 ^c^ ± 0.11	2.70 ^dce^ ± 0.11	5.23 ^e^ ± 0.11	1.00 ^a^ ± 0.00	2.82 ^cd^ ± 0.07	34.56 ^gh^ ± 5.85	18.67 ^de^ ± 4.56	3.67 ^e^ ± 3.50	27.21 ^e^ ± 1.73
V4	1.90 ^d^ ± 0.09	2.37 ^e^ ± 0.09	5.47 ^de^ ± 0.09	0.93 ^a^ ± 0.09	2.92 ^cd^ ± 0.45	299.00 ^e^ ± 5.00	43.349 ^d^ ± 1.550	9.810 ^e^ ± 4.72	84.63 ^a^ ± 1.00
V5	3.75 ^b^ ± 0.15	2.77 ^cd^ ± 0.15	7.87 ^b^ ± 0.15	0.50 ^bc^ ± 0.15	3.05 ^bcd^ ± 0.08	117.33 ^f^ ± 8.33	37.49 ^d^ ± 12.81	15.25 ^e^ ± 4.09	55.68 ^b^ ± 0.34
V6	1.02 ^e^ ± 0.18	2.63 ^de^ ± 0.18	1.03 ^f^ ± 0.18	0.50 ^bc^ ± 0.18	ND	6.22 ^h^ ± 4.81	ND	ND	0.69 ^f^ ± 0.53
V7	1.05 ^e^ ± 0.03	3.07 ^bc^ ± 0.03	6.03^d^ ± 0.03	0.10 ^d^ ± 0.03	2.47 ^d^ ± 0.21	577.89 ^b^ ± 13.47	131.79 ^c^ ± 4.01	114.72 ^b^ ± 11.16	46.72 ^cd^± 2.37
V8	2.15 ^d^ ± 0.08	4.47 ^a^ ± 0.08	7.23 ^bc^ ± 0.08	0.90 ^a^ ± 0.08	5.67 ^a^ ± 0.50	395.10 ^d^ ± 29.6	194.37 ^b^ ± 16.78	89.81 ^c^ ± 2.65	45.72 ^cd^ ± 1.36
V9	4.96 ^a^ ± 0.50	2.70 ^cde^ ± 0.20	7.07 ^c^ ± 0.20	0.50 ^bc^ ± 0.20	6.19 ^a^ ± 0.29	521.22 ^c^ ± 12.73	244.53 ^a^ ± 11.32	225.20 ^a^ ± 17.6	82.18 ^c^ ± 1.49
V10	1.80 ^d^ ± 1.00	2.57 ^de^ ± 0.30	8.67 ^a^ ± 0.12	0.73 ^ab^ ± 0.58	2.58 ^d^ ± 0.32	655.00 ^a^ ± 22.2	105.07 ^b^ ± 21.33	47.81 ^d^ ± 3.31	48.80 ^c^ ± 1.20
V11	0.65 ^e^ ± 0.29	2.80 ^cd^ ± 0.09	1.17 ^f^ ± 0.20	0.27 ^cd^ ± 0.29	ND	82.00 ^fg^ ± 34.67	ND	ND	2.85 ^f^ ± 0.86

Values in the same column with different superscripts are significantly different (*p* < 0.05). ND: not determined.

**Table 2:** Antimicrobial activity of the different vinegars against various pathogens.

Samples ID	*E. coli* ATCC		*E. coli* CIP		*S. aureus* ATCC		*K. pneumonia* ATCC		*C. albicans* ATCC		*A. niger* ATCC	
	DIZ	MIC	DIZ	MIC	DIZ	MIC	DIZ	MIC	DIZ	MIC	DIZ	MIC
V1	17.67 ^bc^ ± 2.52	7.81	17.67 ^b^ ± 0.58	15.62	11.67 ^bcd^ ± 2.89	1.95	20.67 ^cd^ ± 4.04	3.91	34.33 ^a^ ± 0.58	62.5	Rs	Rs
V2	18.00 ^b^ ± 0.00	7.81	12.67 ^bc^ ± 2.52	3.91	14.33 ^abc^ ± 0.58	1.95	29.67 ^ab^ ± 0.58	3.91	26.67 ^bc^ ± 2.89	125	Rs	Rs
V3	18.33 ^b^ ± 1.53	7.81	17.67 ^b^ ± 0.58	7.81	16.00 ^ab^ ± 1.73	1.95	24.67 ^bc^ ± 1.53	31.25	26.67 ^bc^ ± 2.89	250	Rs	Rs
V4	7.33 ^f^ ± 0.58	7.81	7.00 ^cd^ ± 0.00	15.62	9.00 ^d^ ± 1.73	3.91	26.00 ^bc^ ± 1.00	15.63	23.33 ^c^ ± 2.89	62.5	Rs	Rs
V5	13.00 ^de^ ± 1.00	7.81	16.67 ^b^ ± 1.53	3.91	14.00 ^abc^ ± 1.00	1.95	32.67 ^a^ ± 2.52	31.25	29.67 ^ab^ ± 0.58	500	Rs	Rs
V6	6.67 ^f^ ± 0.58	1.95	6.33 ^d^ ± 2.08	15.62	10.00 ^cd^ ± 0.00	3.91	17.67 ^d^ ± 2.52	15.63	23.33 ^c^ ± 2.89	31.25	Rs	Rs
V7	8.67 ^ef^ ± 2.31	7.81	7.00 ^cd^ ± 0.00	62.5	Rs	Rs	10.33 ^e^ ± 0.58	250	8.00 ^e^ ± 0.00	250	Rs	Rs
V8	13.33 ^cd^ ± 2.89	3.9	12.67 ^bc^ ± 6.43	15.62	12.67 ^abcd^ ± 2.31	7.81	31.67 ^a^ ± 1.53	7.81	15.67 ^d^ ± 1.15	62.5	Rs	Rs
V9	18.67 ^b^ ± 1.15	1.95	15.00 ^b^ ± 0.00	1.95	16.33 ^a^ ± 1.15	1.95	29.00 ^ab^ ± 1.00	3.91	30.00 ^ab^ ± 0.00	62.5	Rs	Rs
V10	24.67 ^a^ ± 0.58	3.9	25.33 ^a^ ± 0.58	3.9	14.00 ^abc^ ± 1.00	1.95	22.67 ^cd^ ± 0.58	3.91	Rs	Rs	Rs	Rs
V11	25.67 ^a^ ± 0.58	3.9	Rs	Rs	11.00 ^cd^ ± 1.00	1.95	8.67 ^e^ ± 1.15	31.25	Rs	Rs	Rs	Rs
Voriconazole *	-	-	-	-	-	-	-	-	-	-	12	0.5
Fluconazole *	-	-	-	-	-	-	-	-	21	0.4	-	-
Ampicilline *	Rs	Rs	Rs	Rs	Rs	Rs	Rs	Rs	-	-	-	-
Streptomycine *	Rs	0.25	Rs	0.5	9	Rs	Rs	0.003	-	-	-	-

Values in the same column with different superscripts letters are significantly different (*p* < 0.05). DIZ: diameter of inhibition zone (mm); MIC: minimum inhibitory concentration (µL/mL); * MIC expressed in mg/mL; Rs: resistant.

## Data Availability

Not applicable.

## Sample Availability

Samples are available from the authors upon reasonable request.
